# The Development and Lifetime Stability Improvement of Guanosine-Based Supramolecular Hydrogels through Optimized Structure

**DOI:** 10.1155/2019/6258248

**Published:** 2019-06-13

**Authors:** Miao Chen, Weimin Lin, Le Hong, Ning Ji, Hang Zhao

**Affiliations:** State Key Laboratory of Oral Diseases, National Clinical Research Center for Oral Diseases, West China Hospital of Stomatology, Sichuan University, Chengdu 610041, China

## Abstract

Guanosine is an important building block for supramolecular gels owing to the unique self-assembly property that results from the unique hydrogen bond acceptors and donor groups. Guanosine-derived supramolecular hydrogels have promise in the fields of drug delivery, targeted release, tissue engineering applications,* etc.* However, the property of poor longevity and the need for excess cations hinder the widespread applications of guanosine hydrogels. Although guanosine-derived supramolecular hydrogels have been reviewed previously by Dash et al., the structural framework of this review is different, as the modification of guanosine is described at the molecular level. In this review, we summarize the development and lifetime stability improvement of guanosine-based supramolecular hydrogels through optimized structure and elaborate on three aspects: sugar modification, base modification, and binary gels. Additionally, we introduce the concept and recent research progress of self-healing gels, providing inspiration for the development of guanosine-derived supramolecular hydrogels with longer lifespans, unique physicochemical properties, and biological activities.

## 1. Introduction

Gels play an important role in the composition of organisms. The muscles, skin, cell membranes, and cartilage in the human body can be regarded as gels. They are currently applied in various fields such as medicine, materials science, and pharmacy [1]. The colloidal particles or polymers in the sol or solution are linked to each other by physical or chemical forces under certain conditions to form a spatial network structure, and the structure voids are filled with liquid as dispersion medium; such a special dispersion system is called a gel and often contains a large amount of liquid inside. The process of forming a gel from a solution or sol is called gelation.

**Figure 1:**
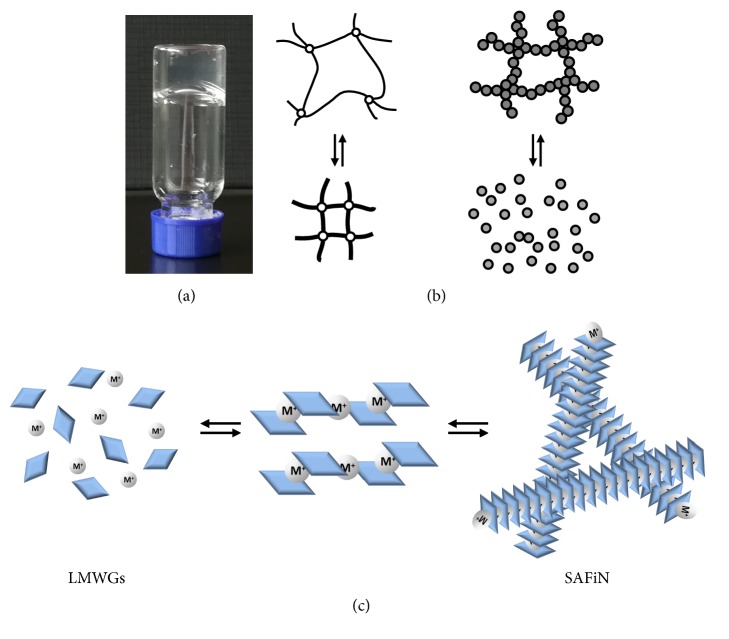
(a) The assessment method of gels is to invert the test tube, and gels are formed if no liquid flows down the tube wall. (b) Chemical gels are three-dimensional (3D) network polymers formed by cross-linking of chemical bonds and are permanent. Physical gels are formed by physical forces such as electrostatic interactions, hydrogen bonding, and chain entanglement, which are nonpermanent and can be converted to solutions by heating the gels. (c) Molecular gelation generally undergoes a nucleation reaction, which is self-assembled by low molecular weight gelators (LMWGs) to form a self-assembled fiber network (SAFiN).

Gels should meet two criteria: One is the rheological standard, where the material can be restored to its original shape when the stress is relieved to a certain degree. Another is being structurally standard, which refers to the majority of the materials being liquids under the microscope, although they behave like solids [2–5]. The method used to assess the behavior involves inverting the test tube, and the gels are formed if no liquid flows down the tube wall (Figure 1(a)) [6].

There are many classification methods for gels, which can be classified according to the types by which 3D networks are bonded and the properties of the medium [1]. For example, gels can be divided into chemical gels and physical gels according to the cross-linking method. The 3D networks of chemical gels are held together by covalent cross-linking to provide a substantially permanent structure. Unless the covalent bonds break, the chemical gels would swell or contract upon exposure to external stimuli instead of degradation. In contrast, the physical gels networks depend on noncovalent bonding, such as van der Waals forces, and hydrogen bonds, so the gels degrade rather than deform under certain external stimuli (Figure 1(b)) [7].

Supramolecular gels generally refer to gels made of “low molecular weight gelators” (LMWGs, molecular weight ≤ 3000 Daltons) dissolved in organic solvents or water and are generally physical gels with good biocompatibility, biodegradability, gel-sol interconversion property, and good drug-loading performance. Gelator molecules undergo one-dimensional (1D) orientation growth through the noncovalent interactions of various structural units, resulting in the formation of linear or band fibers. Then, they overlap to form a 3D network structure, which is commonly referred to as self-assembled fiber networks (SAFiNs) (Figure 1(c)) [8–16].

The study of low molecular weight hydrogel factors can be traced back to 1841, when Lipowitz studied the solubility of uric acid in the presence of lithium carbonate and found that the aqueous solution became a jelly. Most LMWGs are derived from natural products such as amino acids and peptides [13, 17–20], fatty acids [21–23], sugar [11, 23], cholesterol, nuclear bases, nucleosides, and nucleotides [24, 25]. They are often used as supramolecular blocks to produce higher structures owing to their diverse structures, multiple binding sites, self-assembly capabilities, biocompatibility, stability, and stimuli responsiveness [26].

**Figure 2:**
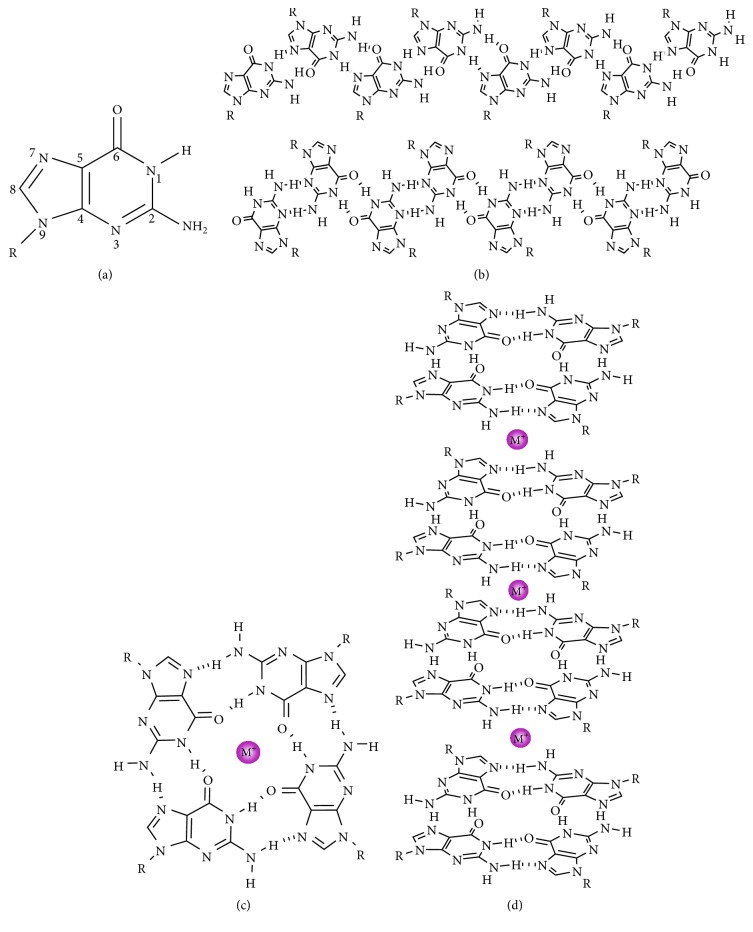
(a) Acceptors and donors of H-bond in guanosine. (b) Two ribbon-like structures of lipophilic guanosine derivatives. (c) G-quartet. (d) G-quadruplex.

Among the five nucleosides, guanosine has unique self-assembly properties because of the unique hydrogen bond acceptors and donor groups, and it is thus an important building block for supramolecular gels. Guanosine (Figure 2(a)) and derivatives form different structures under certain conditions [27], such as a band-like structure (Figure 2(b)), G-quartet (Figure 2(c)), a plane, supported by eight hydrogen bonds, and G-quadruplex (Figure 2(d)), forming a dimer, band, flake, or macrocyclic compounds [28–36].

With the increasing applications of hydrogels, simply improving the mechanical properties of hydrogels is far from meeting the practical requirements, so functional hydrogels have attracted more and more attention. Functional hydrogels can respond to changes in the external environment, such as temperature, pH, pressure, magnetic force, solvent polarity, to achieve gel-sol or swelling morphological changes and functional reversible regulation. In general, when a guanosine building unit is modified, it usually imparts specific functions to the supramolecular gel. In addition, self-assembly forms a supramolecular hydrogel with highly ordered nanostructures, often accompanied by special functions during assembly. Therefore, guanosine-derived supramolecular hydrogels play an important role in functional hydrogels. Owing to guanosine derivatives' unique molecular structure, biocompatibility, and stimuli responsiveness, guanosine-derived supramolecular hydrogels exhibit good potential in drug delivery [37], targeted release, regenerative medicine, environmental remediation, 3D printing, sensors, catalysis,* etc*. [7, 14, 15, 17, 38]. Guanosine-derived supramolecular hydrogels have a 3D network system with space for embedding or containing drug molecules that can be used for drug delivery. Functional hydrogels can respond to changes in the external environment by changing morphology or even self-destruction, achieving the purpose of controlled release and sustained release of loaded drugs. Due to the biocompatibility, extremely low cytotoxicity, and mechanical adjustability of some guanosine-derived supramolecular hydrogels, they can be used as a cell growth matrix or tissue engineering scaffold [39]. And self-healing properties of hydrogels, usually accompanied by injectability, have great application prospects in the fields of tissue engineering, wound dressing, 3D printing, soft robots,* etc*. There are functional groups in the molecular structure of hydrogel that can bind to heavy metal ions or dye molecules to achieve selective removal of contaminants in water [38, 40]. In addition, some guanosine-derived supramolecular hydrogels also exhibit special properties such as fluorescence activity, antibacterial activity, and enzyme activity, further expanding the application prospects of the hydrogels.

## 2. The Discovery Process of Guanosine Assemblies and the Formation of Supramolecular Hydrogels

**Figure 3:**
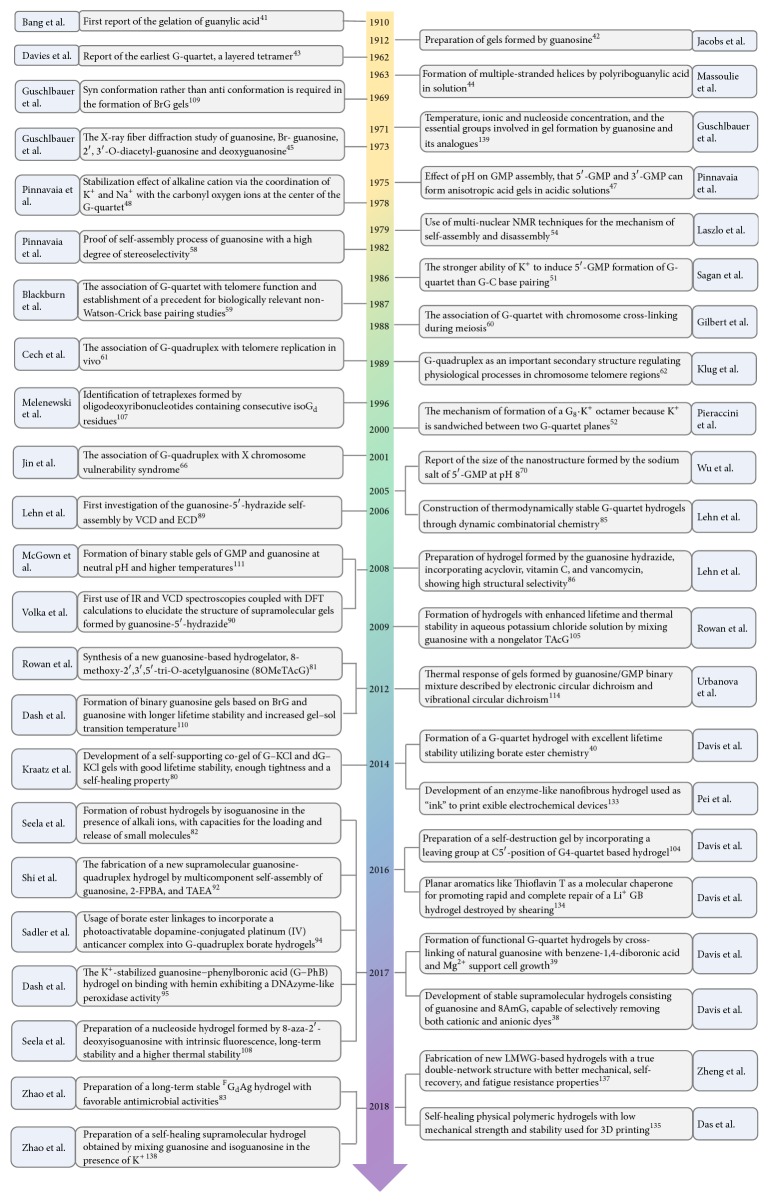
The development and lifetime stability improvement of guanosine-based supramolecular hydrogels [38–45, 47, 48, 51, 52, 54, 58–62, 66, 70, 78, 79, 82, 83, 85, 86, 89, 90, 92, 94, 95, 104, 105, 107–111, 114, 133–135, 137–139].

From the discovery of guanosine hydrogels with two shortcomings, the poor longevity and the need for excess cations, they have been gradually optimized to long-lived and widely used guanosine derivative hydrogels which span more than a century (Figure 3).

In 1910, the gelation of guanylic acid was firstly reported by Ivar Bang, which was considered the first report of guanosine hydrogels. Thus, guanosine and analogs have a long history of forming low molecular weight hydrogel gels [41]. In 1912, gels derived from guanosine were reported by Levene and Jacobs [42].

The earliest G-quartet was determined by performing studies involving hydrogels formed from the sodium salt of 5′-GMP. In 1962, Gellert et al. reported 3′-guanosine monophosphate (3′-GMP) and 5′-guanosine monophosphate (5′-GMP) and utilized hydrogen bonds to form layered tetramers, the earliest G-quartet. From X-ray diffraction experiments, they found that four guanosine molecules form a planar ring G-quartet, which was supported by eight hydrogen bonds, and the stack spacing of the G-quartet was 3.75 A and rotates at a certain angle [43]. Fresco and Massouzi also reported that polyriboguanylic acid can form multiple-stranded helices in solution [44]. In 1972, Guschlbauer et al. reported the X-ray fiber diffraction study of guanosine, 8-Br-guanosine, 2′,3′-O-diacetyl-guanosine, and deoxyguanosine [45].

Guanosine was subsequently discovered to form hydrogels by forming a similar helical arrangement. In subsequent years (the 1970s and 1980s), there were two important discoveries regarding the self-assembly of guanosine [46], namely, the effect of pH on GMP assembly [47] and the stabilization effect of alkaline cation [48]. First, 5′-GMP does not gel at all conditions. Only under acidic conditions, it forms anisotropic acid gels [47]. Secondly, metal cations can bind to the four oxygen atoms of the G-quartet center and are essential for the stabilization of the G-quartet [49, 50]. Only certain alkali metal ions can induce self-assembly of 5′-GMP. K^+^ and Na^+^ coordinate with the carbonyl oxygen ions at the center of the G-quartet to stabilize it, while Li^+^ and Cs^+^ have no such effect.

The ability of K^+^ to induce 5′-GMP formation of G-quartet is stronger than G-C base pairing [51]. Pinnavaia et al. also reported that, because of the large size of K+, it cannot be coplanar with the G-quartet and can only be sandwiched between two planes to form a G_8_•K^+^ octamer [46, 52, 53]. The G_8_•M^+^ octamer combined with cations was considered as the building block to form a cylindrical structure by G-quartet stacking. In order to study the mechanism of self-assembly and disassembly, Laszlo et al. used multinuclear nuclear magnetic resonance (NMR) techniques [54, 55], and NMR then became an important means to study guanosine self-assembly [56, 57]. Two stacked chiral G-quartets can achieve at least six kinds of G_8_•M^+^ nonisomer. However, Na^+^ NMR studies of 5′-GMP indicated that only two stable nonidentical isomers existed. This shows that the self-assembly process of guanosine has a high degree of stereoselectivity [58].

Early studies on 5′-GMP considered the structure, stereochemistry, mechanisms, and functions, whereas in the late 1980s, the G-quartet began to be an area of concern. Results of studies have indicated that G-quadruplex is potentially biologically significant, for example, related motifs for gene expression, fragile X syndrome, and telomerase inhibition. Henderson et al. proposed that the G-quartet was associated with the telomere function and established a precedent for biologically relevant non-Watson-Crick base pairing studies [59]. Gilbert et al. proposed that G-quartet was associated with chromosome cross-linking during meiosis [60]. Williamson et al. proposed that the G-quadruplex is associated with telomere replication in vivo [61]. Sundquist et al. also proposed that G-quadruplex was a crucial secondary structure that regulated physiological processes in chromosome telomere regions [62]. In 1990, Guschlbauer et al. published a review to highlight the fact that G-quadruplex gradually showed its importance [63]. Some specific oligonucleotide sequences of DNA and RNA can form G-quadruplexes in vitro. These sequences also appear in the telomere region of the chromosome, the promoter region and recombination site of the gene, the RNA packaging site, and the RNA dimer domains [64, 65]. Darnell et al. proposed that the G-quadruplex was associated with the X chromosome vulnerability syndrome [66]. In their review, Bryan et al. speculated that G-quadruplex structures may be widely used to control the translation process in gene expression [67]

In recent years, the G-quadruplex structure has attracted strong interest due to its rich biological significance [68, 69]. In addition, the G-quadruplex structure has been accurately characterized by advanced technologies such as solid-state NMR technology [52, 69–77]. Although guanosine-forming gels have a long history, two disadvantages cannot be ignored, that is, the poor longevity and the need for excess cations, especially K^+^ ions, which hinder the potential application of guanosine-based hydrogels in physical and biological applications [7]. In order to address this issue, a series of studies focused primarily on preparing guanosine derivatives to increase the lifetime of hydrogels [78–81].

In this review, we summarize the development and lifetime stability improvement of guanosine-based supramolecular hydrogels through optimized structure and elaborate on three aspects: sugar modification, base modification, and binary gels. Finally, we introduce the concept and recent research progress of self-healing gels to better understand the structure and design of guanosine-based gels and to provide inspiration for the development of guanosine-derived supramolecular hydrogels with a longer lifespan and unique physicochemical properties and biological activities.

## 3. Strategies to Improve the Lifetime of Guanosine-Derived Supramolecular Gels

**Figure 4:**
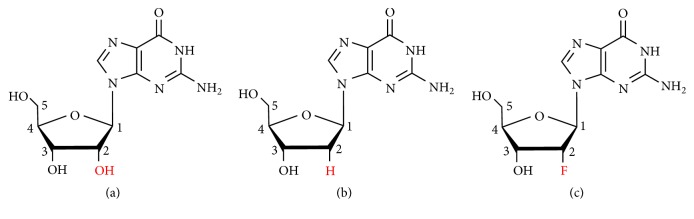
(a) Guanosine (G). (b) 2′-Deoxyguanosine (G_d_). (c) 2′-Deoxy-2′-fluoroguanosine (^F^G_d_).

Guanosine is composed of five-carbon sugar and guanine base (Figure 4(a)). Therefore, guanosine derivatives can be obtained by modifying bases or sugar groups. The G-quartets formed by the coordination between the hydrogen bond acceptor and donor at the guanine base form the basis of hydrogels preparation. In order to avoid the destruction of the G-quartets structure, researchers first tried to modify the guanosine sugar group and then tried to modify the base of guanosine based on protecting the G-quartets to ensure self-assembly properties. Recently, it has also been noted that binary gels formed from different guanosine derivatives have unique properties and enhanced life stability.

### 3.1. Sugar-Modified Guanosine Derivatives

The modification sites of guanosine sugar are positions 2′, 3′, and 5′ (Figure 4(a)). Researchers have modified one or several sites to form new guanosine derivative gelling agents in order to improve the life stability of guanosine hydrogels or to obtain different physicochemical properties or biological activities. In recent years, the sugar-modified guanosine derivative supramolecular hydrogels in recent years are summarized in the following four general modifications: 2′ position independent; 5′ position independent; 2′, 3′ positions combined; and 2′, 3′, 5′ positions combined modifications.

#### 3.1.1. 2′-Deoxyguanosine and 2′-Deoxy-2′-fluoroguanosine

Seela et al. compared the hydrogelation properties of guanosine (G), 2′-deoxyguanosine (G_d_), and 2′-deoxy-2′-fluoroguanosine (^F^G_d_) (Figure 4) [82]. Their findings indicated that the gelation data of G and G_d_ were consistent with existing data performed under slightly different conditions [78]. In this aspect, G behaved like ^F^G_d_, which formed a transparent gel in the presence of K^+^ ions while crystallization occurred in water or the presence of the other alkali metal ions like Li^+^, Na^+^, Rb^+^, and Cs^+^. In the presence of K^+^ ions, the hydrogels formed by three compounds, the T_gel_ (gel melting temperature) values showed the order G> ^F^G_d_ > G_d_, and the lifetime order was as follows: ^F^G_d_ > G_d_ >G. However, three guanosine gels were relatively unstable and had a lifetime of minutes or hours, and fluoride did not have obvious effect on lifetime.

Because the balance conditions between gelation and crystallization of guanosine-derived hydrogels have not been fully explored, which are crucial for designing new tune lifetime stability and mechanical properties, inspired by this, our group studied the crystallization process and the gel properties of 2′-deoxy-2′-fluoroguanosine (^F^G_d_) (Figure 4(c)) hydrogel, and the phenomenon indicated that only in the presence of K^+^, ^F^G_d_ formed a transparent hydrogel. In addition, silver ions can induce ^F^G_d_ to form a supramolecular hydrogel with excellent lifetime stability because it can block the crystallization of ^F^G_d_ [83]. Furthermore, ^F^G Ag hydrogel also exhibited superior antibacterial activity, especially for NOK-SI cells and* Fusobacterium nucleatum*, which contribute to the research and development of antimicrobial drugs and the future applications of drug delivery systems.

This year, in order to explore the mystery of the crystallization process of guanosine derivatives-based hydrogels, Luo et al. studied the crystallization process of ^F^G_d_ hydrogel and found two subprocesses, small flake-like and bigger piece-like crystals [84]. In both processes, base pairing and hydrogen bonding patterns are similar, but there is slight difference in supramolecular levels, i.e., sugar-sugar, base-base, base-sugar interactions.

#### 3.1.2. Guanosine-5′-Hydrazide

**Figure 5:**
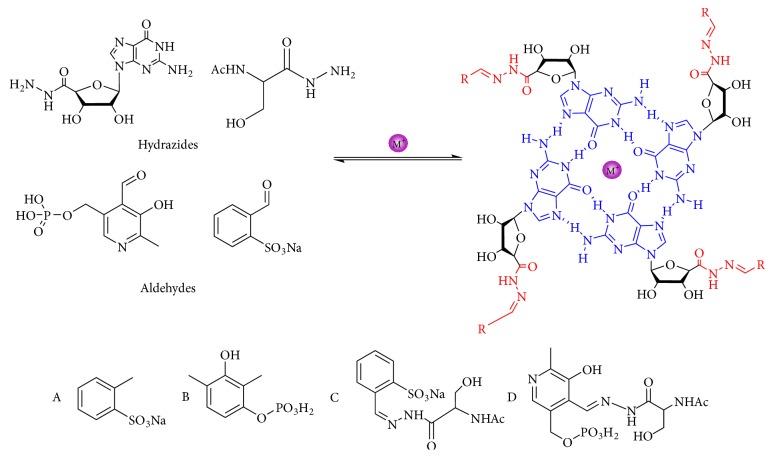
With the presence of metal cations and the formation of a guanine quartet (G-quartet), a stable supramolecular hydrogel is formed from the guanosine hydrazide, among which guanosine-5′-hydrazide is capable of capturing bioactive molecules such as vitamin C, acyclovir, and vancomycin into its hydrogel network.

Recently, hydrogels have attracted some interest in the area of drug release due to the hydrophilicity and potential biocompatibility. Lehn et al. researched the controlled release of small molecules and constructed thermodynamically stable G-quartet hydrogels through dynamic combinatorial chemistry [85]. With the presence of metal cations, a stable supramolecular hydrogel was formed from the guanosine hydrazide, among which guanosine-5′-hydrazide was capable of capturing bioactive molecules such as vitamin C, acyclovir, and vancomycin into its hydrogel networks (Figure 5) [86, 87]. The hydrogels can slowly release the above bioactive molecules by reversible dissociation of acylhydrazone bonds. Thus, this hydrogel can be used as ideal medium for drug delivery and highly selectively controlled release [88]. This work plays a key role in dynamic hydrogels.

In addition, it should be noted that Lehn et al. first investigated the self-assembly of guanosine-5′-hydrazide by electronic circular dichroism (ECD) spectroscopy and vibrational circular dichroism (VCD) [89], providing new information concerning the effects of the medium and temperature as well as the properties of the supramolecular assemblies formed. Then, the molecular structure of a powerful hydrogelator was firstly determined through infrared (IR) and VCD spectroscopies combined with VCD spectral calculations and DFT IR both in a gel and in a solution state [90].

#### 3.1.3. 2′, 3′ Positions Modification

**Figure 6:**
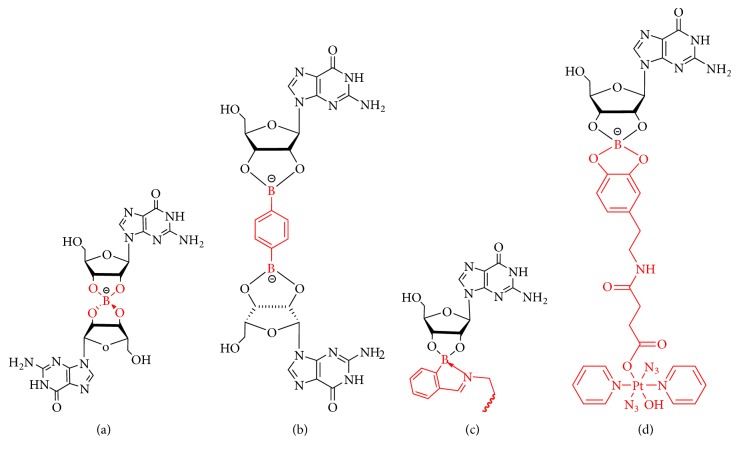
(a) B(OH)4- combined with guanosine produces a guanosine-borate (GB) ester. (b) Natural guanosine is cross-linked with Mg 2+ and benzene-1,4-diboronic acid to form functional G-quartet hydrogels. (c) Self-assembly of 2-formylphenylboronic acid (2-FPBA) and tris(2-aminoethyl)amine (TAEA) in the presence of KCl can prepare G-quartet hydrogels. (d) Photoactivated dopamine-conjugated platinum (IV) anticancer complex (Pt-DA) incorporated into G-quadruplex G4K+ borate hydrogels via borate ester linkages.

As anions can influence the stability and structure of lipophilic G_4_•M^+^ quadruplexes, Davis et al. produced a G-quartet hydrogel with excellent lifetime stability utilizing borate ester chemistry [40, 91], which mainly depends on the anionic, tetravalent borate ester formed as the object of the reaction of B (OH)_3_ or B (OH)_4_ with* cis*-1,2-diols. B (OH)_4_^ ^^−^ combined with guanosine produced a guanosine-borate (GB) ester (Figure 6(a)), and when combined with 0.5 equivalent of potassium borate, guanosine formed a strong self-supporting hydrogel, which exhibited the advantages of a modulus of elasticity>10 kPa and a prolonged lifetime.

In this reaction, the presence of anionic GB diester mainly helps to dissolve guanosine, a recognized insoluble compound, and finally forms GB hydrogel which is more stable and unique in physical properties than other G-quartets gels. The physical properties of the GB hydrogel can be primarily adjusted by changing the cations of the borate. Moreover, the addition of KB (OH)_4_ produces the strongest GB hydrogel, while the replacement with LiB (OH)_4_ produces a weaker material. It was also found that thioflavin T can fluoresce in the presence of G_4_•M^+^ precursor structure.

Natural guanosine is cross-linked with Mg^2+^ and benzene-1,4-diboronic acid to form functional G-quartet hydrogels (Figure 6(b)), which are capable of supporting cell growth without significant gel degradation [39]. Specifically, the water content and structure of the supramolecular hydrogels are determined by the external cross-linking of anion borate by Mg^2+^ and the characteristics of the internal stabilizing cations (K^+^ and Ba^2+^). The specific compositions of hydrogels determine the amount of water that can be incorporated. And the use of Mg^2+^ crosslinker can withstand more than 15 times the incorporation of water. The gel swelling property of hydrogels makes them great candidates for the application of cell growth.

To explore the stimuli-sensitive hydrogels, Shi et al. reported that multicomponent self-assembly of guanosine, tris(2-aminoethyl)amine (TAEA), and 2-formylphenylboronic acid (2-FPBA) in the presence of KCl fabricated a supramolecular G-quartet hydrogel (Figure 6(c)). In addition to being commercially available, the components in this process can be easily self-assembled [92]. The iminoborate bonds can form spontaneously in the accumulation of G-quartets, along with guanosine, TAEA, and 2-FPBA. In addition, the iminoborate bonds, apart from promoting the formation of a hydrogel, also impart glucose and activate its acid responsiveness, which is very useful property for drug control and sustained release. Under the stimulation of glucose and acid, the surface is peeled off and the hydrogel is dissolved, thereby achieving zero-order release of methylene blue and FITC-lysozyme. This supramolecular G-quartet hydrogel formed by small molecule multicomponent self-assembly has strong commercial availability and rich functions and exhibits glucose and pH responsiveness that contribute to zero-order drug release behavior. The application prospects in the biological field are prosperous.

Recently, injectable hydrogels have proven to be a promising candidate as a delivery vehicle for cancer therapy [93]. The application of photochemotherapy in cancer has the potential to provide temporal and spatial constraints on the drug activity in tumors, without obvious side effects on normal cells. Therefore, Sadler et al. incorporated a photoactivated dopamine-conjugated platinum (IV) anticancer complex (Pt-DA) into G_4_K^+^ borate hydrogels (Figure 6(d)) using boronate linkages (Pt-G_4_K^+^B hydrogel) [94]. Pt-G_4_K^+^B hydrogels showed selective phototoxicity to cancer cells* vs* normal fibroblasts (MRC5), which was stronger than the phototoxicity of platinum (IV) anticancer complex (Pt-DA). They adopted a new method to prepare hydrogels that deliver photoactivated platinum (IV) anticancer complexes to cancer cells, while discovering that the chemical modification significantly improved the selectivity between normal cells and cancer cells (more than 18-fold). Such a high potency and selectivity indicate the potential of Pt-G_4_K^+^B hydrogel acting as cancer photochemotherapeutic agents, and it may be used for the locally targeted immunotherapy of cancers.

**Figure 7:**
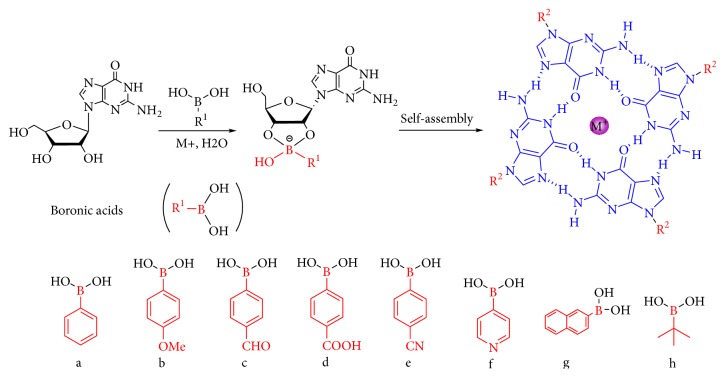
Different types of guanosine-boronate ester hydrogels for biomolecular logic operations and sensing.

To further study anion-stabilized guanosine hydrogels by utilizing anticancer drugs delivery, Dash et al. produced the hydrogel formed by guanosine and boric acid in the presence of Pb^2+^ and K^+^ ions (Figure 7) [95]. Unusually, the K^+^ guanosine-phenylboronic acid (G-PhB) hydrogel exhibits DNAzyme-like peroxidase activity upon binding to hemin [96–103] and promotes oxidation of the 3′,3′,5′,5′-tetramethylbenzidine in the existence of H_2_O_2_. This study demonstrates that peroxidase enzymes can be mimicked by guanosine-derived hydrogels and nanomolar concentrations of lead ions can be detected by hydrogels. In addition, the nontoxic property of K^+^ stabilized G-PhB hydrogel makes it useful for the delivery of anticancer drugs. This approach provides a multifunctional material platform for preparing hydrogels with adjustable elasticity for a variety of applications such as targeting drug delivery, biomolecular computing and sensing.

#### 3.1.4. 2′, 3′, 5′ Positions Modification

**Figure 8:**
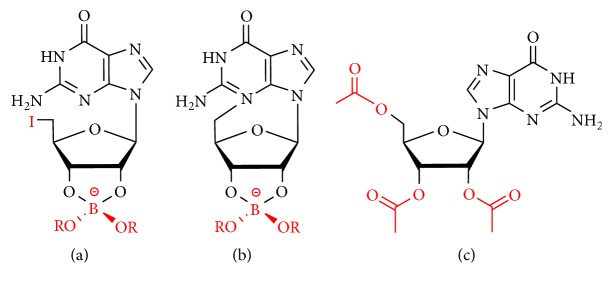
(a) 5′-Deoxy-5′-iodoguanosine (5′-IG) borate esters. (b) 5′-cG. (c) Acetyl derivative 2′,3′,5′-tri-O-acetylguanosine (TAcG).

To achieve the goal of creating a hydrogel that can be used to control the release of preloaded compounds, Davis et al. found that the G_4_-quartet-based hydrogel formed by self-assembly of 5′-deoxy-5′-iodoguanosine (5′-IG) borate esters (Figure 8(a)) undergoes a cyclization process to yield 5′-deoxy-N3,5′-cycloguanosine (5′-cG) (Figure 8(b)), causing the gel to self-degrade [104]. The self-degradation of the hydrogel can be used to release guanine analogs that have been preincorporated into gels, such as the antiviral drugs acyclovir and ganciclovir. Most importantly, this hydrogel has greater drug release property and higher incorporation than the previously reported cationically stabilized guanosine hydrogel systems [86, 87]. This study establishes a new approach to the design of guanosine hydrogel systems for drug delivery and controlled release.

Since crystallization in the gel is a key barrier to the innovative development of a guanosine hydrogel, we assume that adding a nongelating comonomer to guanosine would provide adequate structural heterogeneity to inhibit crystallization. Furthermore, if the nongelling comonomer contains a hydrophobic group that promotes physical (hydrophobic) cross-linking between the guanosine stacks, the guanosine hydrogel can be further imparted with additional thermal stability. Therefore, Rowan et al. found that the hydrophobic guanosine derivative 2′,3′,5′-tri-O-acetylguanosine (TAcG) shown in Figure 8(c), which is commercially available, did not gel in aqueous potassium chloride itself, but can be used to form a stable binary gel when mixed with guanosine [105].

### 3.2. Base-Modified Guanosine Derivatives

#### 3.2.1. Isoguanosine

**Figure 9:**
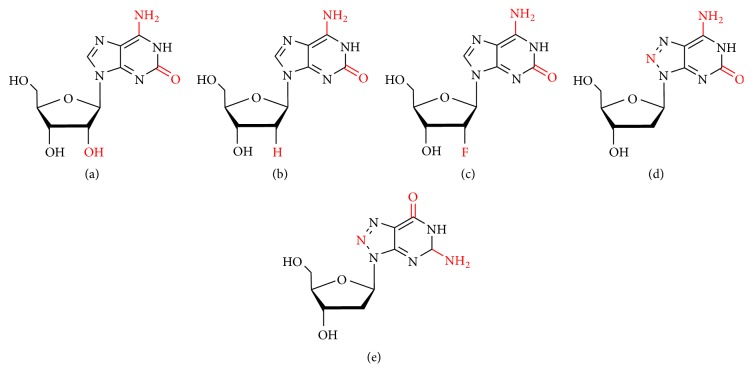
(a) Isoguanosine (isoG). (b) 2′-Deoxyisoguanosine. (c) 2′-Deoxy-2′-fluoroisoguanosine. (d) 8-Aza-2′-deoxyisoguanosine (z^8^isoG_d_). (e) 8-Aza-2′-deoxyguanosine (z^8^G_d_).

Isoguanosine (isoG) is a guanosine (G) isomer with a transposition at the C2 and C6 (Figures 9(a)–9(c)). More than 40 years ago, D. Shugar described an isoG with a quartet structure for the preparation of poly-isoG [106]. About 20 years later, Seela et al. reported the results obtained by identifying tetraploids formed from oligodeoxyribonucleotides containing consecutive isoG_d_ residues and described how they were separated from single-stranded molecules [107]. The rheological data obtained in 2017 indicated that the hydrogel stability of isoG is up to 15 times better than guanosine. In general, isoG gels can be stabilized over a wide pH range (from pH=3 to pH=10), which can last up to several months, while guanosine hydrogels can last for only a few minutes and will disintegrate within a few hours. At the same time, the storage modulus of isoG (1.26 × 10^4^ Pa at 100 Hz) is much higher than that of G (8.78 × 10^2^ Pa at 100 Hz), indicating that the isoG gel is more stable than G. Scanning electron microscopy (SEM) measurements show that the isoG gel has a microscopic structure of interconnected spiral fiber cylinders, while G is a flat ribbon-like microscopic morphology. This difference may explain why isoG hydrogel is much more stable than G hydrogel [82].

Additionally, Seela et al. investigated the rheological properties of a hydrogel prepared by isoG, 2′-deoxyisoguanosine and 2′-deoxy-2′-fluoroisoguanosine (Figures 9(a)–9(c)) under alkaline conditions. Rheological properties include gel crystallization, lifetime stability, temperature of gel melting, and minimum gelation concentration. The results showed that the dependence of the above three nucleosides on alkali metal ions was different, indicating that the hydrophobic 2′-substituent had a certain effect on gel formation (F>H>OH). Therefore, under the physiological conditions simulated by PBS buffer, isoG can form a long-lasting stable hydrogel with excellent release and loading ability of small molecules. Therefore, isoG shows high bioactivity and excellent gel formation capability, having a very broad application prospect in the field of drug delivery and nanodevice construction.

To further explore the fluorescence properties of isoG-derived hydrogels, Seela et al. described a nucleoside hydrogel formed by 8-Aza-2′-deoxyisoguanosine (z^8^isoG_d_) (Figure 9(d)). This new compound represents the first intrinsic-fluorescence-emitting guanosine hydrogel, which responds to external stimuli including pH change, the addition of alkali metal ions, UV irradiation, and heat [108]. Meanwhile, the nucleoside 8-Aza-2′-deoxyguanosine (z^8^G_d_) (Figure 9(e)) forms a solid nanotubular structure without forming a hydrogel. Therefore, the hydrogel formed by z^8^isoG_d_ may be applicable in various fields of nanomedicine and nanobiotechnology.

#### 3.2.2. 8′ Position Modification

**Figure 10:**
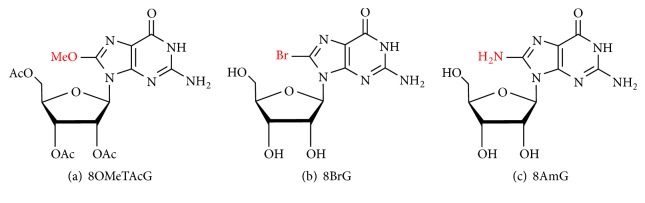
(a) 8-Methoxy-2′,3′,5′-tri-O-acetylguanosine (8OMeTAcG). (b) 8-Bromoguanosine (8BrG). (c) 8-Aminoguanosine (8AmG).

Researchers have also modified the 8′ position of the base to investigate the influence of changes in conformation of guanosine and other nucleosides on gels formation. Guschlbauer et al. investigated the condition which is suitable for BrG gelation, and the optical test results of BrG gels indicate that the* anti-conformation* hinders the process of gelation, while the* syn*-conformation does not [109]. In addition, 8-methoxy-2′,3′,5′-tri-*O*-acetylguanosine (8OMeTAcG) [79], 8-bromoguanosine (8BrG) [110], and 8-aminoguanosine (8AmG) [38] (Figure 10) were also prepared as components of binary gels, which show long-lived stability, along with other specific properties.

### 3.3. Binary Gels

Studies have found that the shortened life expectancy due to gel components precipitation and the requirement for a high concentration of stable cationic environment are major drawbacks of most guanosine derivative hydrogels. To overcome these shortcomings, an adjustable stable gel has been formed, and various studies have recently reported a variety of binary gels formed from two different guanosine derivatives. They usually have unique physical properties while at the same time improving lifetime and enhancing stability by hindering the crystallization of the gel components.

**Figure 11:**
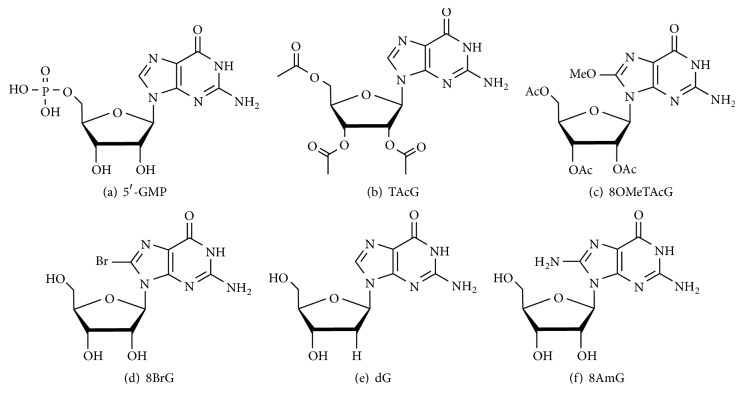
Guanosine derivatives used for the preparation of binary hydrogels. (a) 5′-GMP. (b) 2′,3′,5′-Tri-O-acetylguanosine (TAcG). (c) 8-Methoxy-2′,3′,5′-tri-O-acetylguanosine (8OMeTAcG). (d) 8BrG. (e) dG. (f) 8-Aminoguanosine (8AmG).

In 2008, McGown et al. confirmed that a binary mixture of 5′-guanosine monophosphate (5′-GMP) (Figure 11(a)) and guanosine forms a stable gel under neutral conditions [111]. The stable hydrogel formed by the mixture is attributed to 5′-GMP helping dissolving guanosine. In turn, the insolubility of guanosine contributes to the gelation process of 5′-GMP at low concentrations. The temperature range can be changed when hydrophilic GMP and hydrophobic guanosine are adjusted in relative proportions.

In general, acidic conditions and low temperatures favor gel formation, while these binary mixtures also exhibit thermoassociative behavior. These solutions turn sharply into gels at higher temperatures and are only in a liquid state at the refrigerator temperature (5°C). Additionally, thermoassociative behavior can also be achieved in a binary mixture containing hydrophobic guanosine and a hydrophilic guanosine derivative. This unique phenomenon should be attributed to the role of guanosine in GMP aggregates. The incorporation of guanosine promotes dissolution while reducing the repulsive force between anionic GMPs, stabilizing higher aggregates, thereby promoting liquid crystal gels formation. Finally, it should be noted that the temperature required to form and maintain a stable liquid crystal gel phase will increase as the total concentration of the guanosine compounds increases, demonstrating that the binary mixture of guanosine compounds exhibits unique thermal responsiveness and is tunability linked with ratio, cation, or pH, providing possibility for the exploitation of simple inexpensive materials in biomedical applications.

Over the subsequent year, Rowan et al. reported that a well-known gelator (guanosine) mixed with a nongelator with similar structure to 2′,3′,5′-tri-O-acetylguanosine (TAcG) (Figure 11(b)) can form hydrogels in potassium chloride solution. The lifetime and thermal stability have been significantly improved compared to the hydrogels obtained with guanosine alone [105, 112]. This study found that thermal stability and stiffness of the gel can be regulated by changing the gelation ratio of guanosine: TAcG. In addition, the long life stability, excellent thermomechanical property, and adjustability of these gels are due to the increased hydrophobicity by the addition of TAcG, which reduces the driving force of crystallization, along with an increase in fiber width and a reduction in fiber length.

And in 2012, they further reported a fresh guanosine-based hydrogelator, 8-methoxy-2′,3′,5′-tri-O-acetylguanosine (8OMeTAcG) (Figure 11(c)) [79]. These binary gels based on 8OMeTAcG can be easily formed in cell culture medium (2 wt%) and saline solution (0.5 wt%) at low gelator concentrations and have the advantages of extremely low cytotoxicity, facile injectability, and mechanical adjustability (the modulus and yield stress can be adjusted by using different proportions of comonomer). These advantages make the 8OMeTAcG-based co-gel a new class of tissue engineering scaffolding material [113].

In the same year, a study was conducted using 8-bromoguanosine (8BrG) and guanosine to prepare a binary gel (Figure 11(d)) [110]. This hydrogel is opaque and begins to precipitate within 48 hours, compared to a fast-precipitating transparent gel formed with excess KCl and guanosine. However, a binary mixture of guanosine and BrG can form a transparent and stable hydrogel that exhibits superior lifetime stability and higher temperature of gel-sol transition. In addition, this study has confirmed that small molecule dyes can be effectively diffused into this gel and released, fully demonstrating that the gel may be used as a drug delivery vehicle.

As for the self-assembly system, we mainly determine its characteristics by IR, Raman, NMR, SEM, fluorescence spectroscopy, and transmission electron microscopy (TEM). However, recently, a new method such as circular dichroism (CD) technology has been adopted. Urbanová et al. used ECD and VCD to study the responsiveness of the guanosine/GMP mixture to concentration, temperature, and molar ratio [114]. It should be noted that GMP helps to solubilize guanosine, a molecule that has poor solubility in water, and the addition of guanosine promotes the gelation of GMP in water. The binary hydrogel exhibits unique thermoresponsive and thermoassociation properties and has potential application in catalysis, sensors, and drug delivery.

Guschlbauer et al. prepared transparent self-standing supramolecular hydrogels using potassium-ion-mediated self-organization of 8-bromoguanosine and guanosine [111], in which individual components precipitated within several hours. Small molecule dyes represented by rhodamine-6-G, rose bengal, and fluorescein can diffuse through the hydrogel network to form birefringence on the surface, followed by controlled release, demonstrating the potential as a drug delivery medium, as an optical device, and for biomolecular imaging.

In 2014, the latest research of Kraatz et al. showed that a self-supporting tight gel prepared by guanosine had poor life stability in the presence of KCl. In contrast, the gel formed by dG in the presence of KCl was very loose, while it could be maintained for more than 4 months and exhibited excellent thixotropy. Therefore, G-KCl gel and dG-KCl gel are not applicable if there is a high requirement for tightness or life stability in practical applications. In response to this situation, they attempted to design a self-supporting co-gel system by simply mixing equimolar amounts of G-KCl and dG-KCl (Figure 11(e)) gels [78]. Experimental results showed that this co-gel combined the advantages of both, with considerable tightness, sufficient life stability, self-healing, and injectable properties. Interestingly, the morphology, fluorescence, and rheological property of the gels changed significantly when induced by other monovalent cations.

In 2017, Davis et al. developed a stable, transparent supramolecular hydrogel consisting of a binary mixture of 8-aminoguanosine (8AmG) and guanosine (Figure 11(f)) with either K^+^ or Ba^2+^ salts. This study suggests that a large amount of 8AmG will be protonated in a G_4_-hydrogel, and the cationic network present in the hydrogel can enhance electrostatic binding to the anionic dye. In addition, the 8AmG/G-Ba^2+^ gel has a stronger binding ability to the anionic naphthol blue black than the 8AmG/G-K^+^ hydrogel and is stiffer than the K^+^ hydrogel. This gel is capable of selectively removing the cation [40] and anion [38] dyes from aqueous solutions and can alter the charges by changing the gel-forming salts, providing a simple and versatile method for removing contaminating dyes.

## 4. Self-Healing Supramolecular Guanosine-Derived Hydrogel

Self-healing is a rare property of nucleoside-based gelators, but it is desirable to establish injectability of clinic gel materials and to allow a wide range of applications. It represents the ability of materials to improve the lifetime and spontaneously repair their damage, which is an important function in the organisms. It is one of the emerging fields to research the development of self-healable supramolecular gels. Self-healing property means when a piece of gel is cut into several pieces and then the pieces join together, they are combined into one continuous piece. LMWG-based self-healing organogels [115–121] and hydrogels [122–129] have been recently reported.

Therefore, it is highly desirable to print electronic devices directly into designed shapes and tailored sizes with in vivo/vitro biocompatibility, so that the special demands such as soft robotics [130], medical devices [131], and tissue engineering [132] can be satisfied. Therefore, Pei et al. developed a new enzyme-like nanofiber hydrogel in the cationic templated self-assembly process between guanosine and KB (OH)_4_ by adding hemin to the G-quartet scaffold, and further the enzymatic mimetic function was used to semiconductorize the H/G_4_-PANI hydrogel, which is used as the “ink” to print plastic electrochemical devices [133].

Today, there is growing interest in the capability of molecular chaperones to regulate the properties and structures of supramolecular gels. In the study, Davis et al. found that thioflavin T (ThT) acted as a molecular chaperone to expedite the gelation, increase the hardness of Li^+^ guanosine-borate (GB) hydrogel, and promote the relatively complete and rapid repair of sheared Li^+^ GB hydrogels [134]. In the presence of Li^+^, guanosine tends to form ribbon-like aggregates and produce a weak gel. The Li^+^ GB hydrogel is hardened by ThT stabilizing the G_4_–quartet structure in the fiber network.

In addition, other planar aromatic hydrocarbons such as crystal violet (CV), methylene blue (MB), and thiazole orange (TO) also increase the rigidity of hydrogels. This study suggested that ligands that bind to G_4_-quartets also act as molecular chaperones to promote the preparation of guanosine-based hydrogel assemblies with enhanced lifetime stability.

As gels produced by dG show enhanced properties such as mechanical reversibility and lifetime stability, Kraatz et al. simply mixed equimolar amounts of dG-KCl and G-KCl gels and succeeded in engineering the development of self-supporting co-gels [78]. This co-gel showed extraordinary self-healing property, sufficient hardness, and lifetime stability without any clue of crystallization. Rheological data showed that the original viscosity of the co-gel could be fully recovered no more than 15s after damage, indicating its excellent self-healing property. It is fair enough to make a conclusion that the instantly healing characteristic as well as the shear-thinning behavior ensures that it as a preferable choice for injectable scaffolds, which is desirable for clinical gel materials.

Self-healing physical polymeric hydrogels with low mechanical stability and strength can be used for 3D printing [135, 136]. The 3D printing process is driven by self-healable and highly thixotropic property as well as the injectability of the gel, providing inspiration for 3D bioprinting in the application of low-molecular-weight-biocompatible hydrogels.

However, a majority of supramolecular hydrogels based on LMWG are extremely mechanically weak. They cannot even bear conventional tearing and tensile tests. Recently, a group focused on fabricating a new LMWG-based hydrogel (G_4_·K^+^/PDMAAm DN gel) with a true dual network structure. The first chemical network is the supramolecular self-assembly of guanosine, KOH and B (OH)_3_, the second is physical G_4_·K^+^ network and the covalently cross-linked poly(N,N′-dimethylacrylamide) (PDMAAm). This gel is superior to LMWG-based hybrid double network hydrogels (DN gels) in terms of mechanical, fatigue resistance, and self-healing properties [137]. A new avenue is provided for the development of mechanically stiff and multifunctional LMWG-based hydrogels, based on their design principles along with the new G_4_·K^+^/PDMAAm DN gel system.

**Figure 12:**
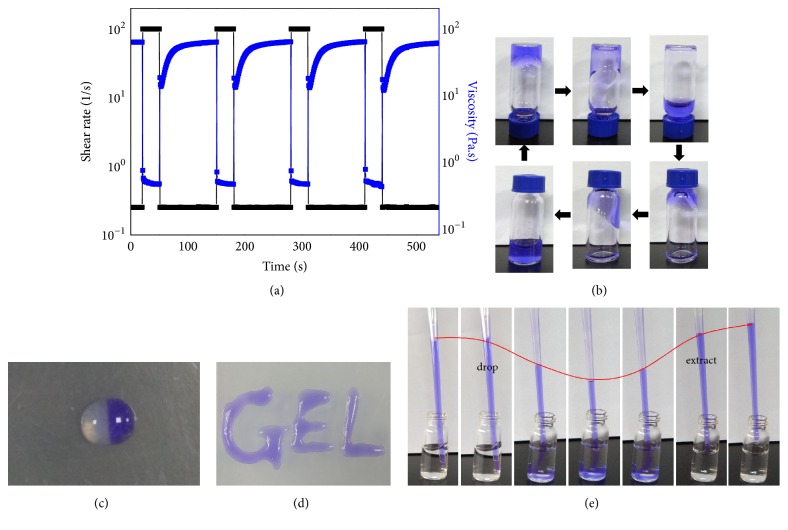
(a) Recovery test of the G/isoG co-gel; blue and black lines represent the shear rate and viscosity, respectively. (b) Photos showing the self-healing property found in co-gel. (c, d) Excellent viscosity and writing ability of the mixture. (e) The mixture was extracted completely with a dropper after dropping into a solution containing K^+^.

This year, a self-healing supramolecular nucleoside hydrogel was obtained by us through mixing guanosine (G) and isoguanosine (isoG) in the K^+^ solution [138]. As far as we know, it is indeed the very first sample of self-healing nucleoside hydrogel based on isoG. The recovery test was repeated for four cycles of shear and recovery. The crosslinks of the co-gel were destroyed after it was submitted to a mechanical loading with the 100 shear rate, but the original viscosity could be completely recovered after releasing the high strain at the 0.25 shear rate for 100s, demonstrating the excellent self-healing characteristic of this co-gel, which is clearly presented in Figure 12.

This co-gel shows a good writing ability as well as viscosity, which allows it to be injected through needles with different sizes without collapsing or blocking. What is more, it could also be completely extracted with a dropper after dropping into a solution containing K^+^, indicating its status as a popular candidate in many fields in the future.

## 5. Summary

This review has detailed the development history and lifetime stability improvement of guanosine-based supramolecular hydrogels through optimized structure. Researchers tried to modify sugar or base groups and introduced binary gels to overcome the previous defects of poor longevity and the need for excess cations. The rare self-healing property accompanied by injectability improves the lifetime of supramolecular hydrogels. As the structure and design of guanosine-based supramolecular hydrogels become better investigated, it is expected that there will be more studies in the future aimed at preparing guanosine-based supramolecular hydrogels with longer lifespan, unique physicochemical properties, and biological activities for a broad range of biological and physical applications.

## Supplementary Materials

Supplementary Materials Graphical Abstract: Despite the long history of guanosine hydrogels, poor longevity and the need for excess cations hinder the potential application in biological fields. To address this issue, the structure of guanosine has been gradually optimized by sugar or base modification, and chemical structures modification plays an important role in forming lifetime stable guanosine-based hydrogels with modulable properties.
