# Supplementary material for: The Development and Lifetime Stability Improvement of Guanosine-Based Supramolecular Hydrogels through Optimized Structure
Source: Biomed Res Int. 2019 Jun 13;2019:6258248. doi: 10.1155/2019/6258248 (PMC6595390; doi:10.1155/2019/6258248)
Supplement: Supplementary Materials — Graphical Abstract: Despite the long history of guanosine hydrogels, poor longevity and the need for excess cations hinder the potential application in biological fields. To address this issue, the structure of guanosine has been gradually optimized by sugar or base modification, and chemical structures modification plays an important role in forming lifetime stable guanosine-based hydrogels with modulable properties. [file 6258248.f1.docx]

**Graphic Abstract**

**
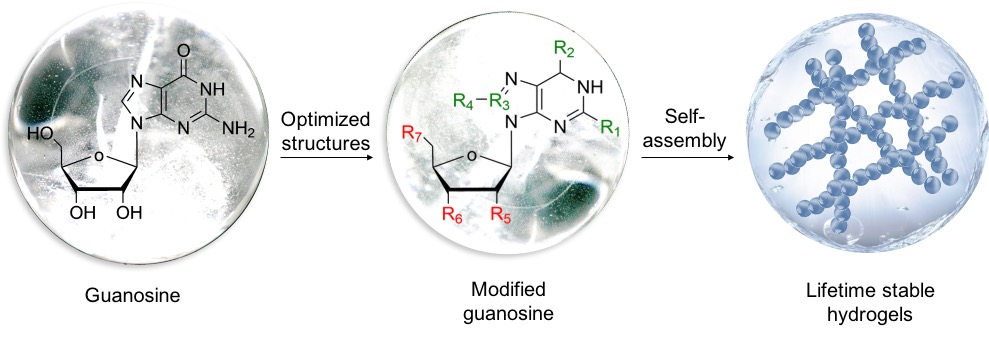
**

Despite the long history of guanosine hydrogels, poor longevity and the need for excess cations hinder the potential application in biological fields. To address this issue, the structure of guanosine has been gradually optimized by sugar or base modification, and chemical structures modification plays an important role to form lifetime stable guanosine-based hydrogels with modulable properties.
